# Plant Resistance to Abiotic Stresses

**DOI:** 10.3390/plants8120553

**Published:** 2019-11-28

**Authors:** Maria-Cecilia D. Costa, Jill M. Farrant

**Affiliations:** 1 Department of Plant Sciences, Technical University of Munich, 85354 Freising, Germany; 2 Department of Molecular and Cell Biology, University of Cape Town, Cape Town 7700, South Africa; jill.farrant@uct.ac.ya

**Keywords:** water stress, high salinity stress, heat stress, orphan crop, nickel hyper-accumulation

## Abstract

Extreme weather events are one of the biggest dangers posed by climate breakdown. As the temperatures increase, droughts and desertification will render whole regions inhospitable to agriculture. At the same time, other regions might suffer significant crop losses due to floods. Usually, regional food shortages can be covered by surpluses from elsewhere on the planet. However, the climate breakdown could trigger sustained food supply disruptions globally. Therefore, it is necessary to develop more stress-resilient crop alternatives by both breeding new varieties and promoting underutilized crop species (orphan crops). The articles in this special issue cover responses of staple crops and orphan crops to abiotic stresses relevant under the climate breakdown, such as heat, water, high salinity, nitrogen, and heavy metal stresses. This information will certainly complement a toolkit that can help inform, support, and influence the design of measures to deal with the climate crisis.

There is a general consensus that global heating is occurring, with predictions that average surface temperatures will increase by roughly 0.2 °C per decade over the next 30 years. This, in turn, is predicted to result in increased frequencies of extreme weather events, drastically reducing crop yields [1]. This is already in evidence in the altered start and end of growing seasons over the past 20 years, which has contributed to regional crop yield reductions, reduced freshwater availability, and has exacerbated the loss of biodiversity [2]. These effects are not uniformly distributed among regions, countries, and land-management systems. It is proposed that European Mediterranean countries are likely to experience more frequent droughts and warmer summers, and temperature increases in North America will result in a shift in the start of the rainy season from spring to winter [3]. El Niño events might grow more frequent, bringing more rain to South American coastal areas and less rain to Indonesia, the Pacific Rim, and Australia [3]. The severity of monsoon rains in Asia might increase, causing more floods in the lowlands [3]. Warmer temperatures will speed up the melting of permafrost in Siberia and increase desertification in Northern Africa [3]. These factors, in combination with elevated salinity in groundwater, poor agricultural practices, and human-related disturbances, are resulting in a considerable reduction of agricultural yield and, thus, local (and inevitably global) food insecurity. This Special Edition reports on evidence of the effects of such abiotic stressors on select crops, and we summarize potential solutions to mitigate food security as a consequence of abiotic stressors.

As one of the primary aspects of climate change, heating itself has a direct bearing on plant mortality. Studies show that global cereal production was reduced by 6.2% between 2000 and 2007 due to the effects of heat and, consequently, drought [4]. In this special issue, Chovanccek et al. (2019) show that heat waves decrease photosynthetic activity and stomatal conductance in wheat. Interestingly, after control conditions were reestablished, plants showed persistent reductions in photosynthesis, suggesting the action of a protective mechanism to prevent the collapse of photoprotective functions [5]. Malerba and Cerana (2019) show that selenium reduces cell death associated with heat stress in tobacco cell cultures by reducing the accumulation of reactive oxygen species, peroxidation of membrane lipids, programmed cell death, and accumulation of stress-related proteins (i.e., BiP, 14-3-3, cytochrome c) [6]. 

Drought has always been the major cause of crop loss, and as discussed above, this is being exacerbated by global heating. In wheat, drought stress leads to increased grain protein content, but with a yield penalty [7]. In this issue, Elbasyoni et al. (2018) utilizes wheat genetic resources to identify loci correlated with grain protein content under drought stress and well-watered conditions. They identify loci that can aid the development of marker-assisted selection for grain protein content, minimizing the yield penalty and leading to a better understanding of the genetic architecture that controls these traits in wheat [7].

Barley is a staple cereal grown in temperate climates globally. High-yielding cultivars generated through breeding have lost tolerance to drought, salt, and heavy metal stress present in the wild ancestor *Hordeum spontaneum* [8]. Stress-tolerant barley landraces from the semi-arid Mediterranean region represent an important source of genetic diversity for the improvement of high-yield cultivars. In this special issue, Landi et al. (2019) investigates how different barley landraces respond to salinity and osmotic stress. Their results can be useful for breeding strategies to reintroduce stress tolerance in cultivated varieties [8]. 

Recently, there has been increased interest in the use of orphan crops, considered to be minor on a global scale, yet important locally for food security in the developing world [9]. Most such crops have greater inherent stress tolerance or resistance than current staple cereals (wheat, rice, and maize) in which such characteristics have been lost by conventional breeding for seed size and yield under well-watered conditions [10]. They are, thus, good candidates to include in molecular breeding programs. However, in many instances, the lack of genomic resources on such species has hindered progress in this regard. Quinoa is an Andean orphan crop adapted to a wide range of marginal agricultural areas, including those prone to drought and high soil salinity. In this special issue, Hinojosa et al. (2018) review the resources available on this species in relation to its tolerance to abiotic stressors [11]. 

Excessive rainfall as a consequence of global heating is also an increasing threat to food security. It has been reported to be the second-largest contributor to loss of maize yields in the United States, totaling damage of 10 billion USD from 1989 to 2016 [12]. Flooding creates low oxygen environments at the root level and causes an imbalance between the slow diffusion of gases in water compared to air and high consumption by microorganisms and roots, leading to oxygen-starved plants [13]. In this issue, Butsayawarapat et al. (2019) characterize mechanisms controlling water-logging tolerance in the orphan crop *Vigna vexillata* (also known as zombi pea or tuber cowpea) [13]. Zombi pea is closely related to cowpea and is cultivated for their fleshy tubers and mature seeds. One of the mechanisms identified is the increased expression of aquaporins, proteins that represent an important selective pathway for water to move across cellular membranes.

Because weather extremes are clearly impacting crop yield, it is also important to take alternative measures to meet the target of maintaining global average temperatures below 2 °C above pre-industrial levels. This requires several actions already in place and additional non-climate actions that can deliver a climate benefit, such as a significant reduction of nitrogen (N) and heavy metal pollution [14]. N pollution has become a major contributor to environmental stresses this century, its main source being an inefficient use and management of synthetic fertilizers and manure by the agricultural sector [14]. Therefore, it is urgent to understand plant responses to N fertilization and seek ways to improve plant N uptake. In this special issue, Wang et al. (2019) investigate the molecular responses of wheat seedlings grown under low N conditions. Their results can be useful to understand the lower limits of N input tolerated by staple crops and for the design of innovative approaches to agricultural N management [15].

Heavy metal pollution might be aggravated by a climate breakdown due to the release of these metals from sediments to water reservoirs [16]. Remediation of heavy metals by conventional methods is challenging and generates several secondary wastes. On the other hand, bio-remediation using plants and microorganisms represents a more promising solution. Bio-remediation involves the adsorption, reduction, or removal of contaminants from the environment by hyper-accumulator plants and microorganisms [16]. In this special issue, van der Pas and Ingle (2019) review the molecular basis of nickel hyper-accumulation in plants and suggest potential future avenues of research in this field [17]. Although nickel is essential for plant growth, extremely high soil concentrations have left farmlands unsuitable for agriculture. Therefore, a better understanding of its functional roles, as well as hyper-accumulation mechanisms, is essential for the formulation of bio-remediation strategies.

The climate crisis is already causing yield losses of major crops and the reduction of arable land worldwide. Hence, to meet our goals of food security, we need to increase the production of food crops that can withstand the ongoing and future environmental changes. This must be done by both developing new varieties of current staple crops and the use of alternative (orphan) crops that produce a harvestable yield in arid and semi-arid regions. At the same time, we must mobilize a wide range of public and private actors to implement measures that reduce emissions, strengthen initiatives already underway, and mitigate the climate crisis. We hope that the articles in this special issue will complement the set of tools that can help inform, support, and influence the design of such measures.
